# Tribological and Vibration Properties of Three Different Polymer Materials for Water-Lubricated Bearings

**DOI:** 10.3390/ma13143154

**Published:** 2020-07-15

**Authors:** Kepeng Wu, Guangwu Zhou, Xiongwei Mi, Ping Zhong, Wenbo Wang, Daxin Liao

**Affiliations:** 1 School of Aeronautics and Astronautics, Sichuan University, Chengdu 610065, China; wukepeng@stu.scu.edu.cn (K.W.); 2017226210010@stu.scu.edu.cn (X.M.); liaodaxin@stu.scu.edu.cn (D.L.); 2 Industrial Turbine Division, Dongfang Turbine Co., Ltd., Deyang 618000, China; zhongping@dongfang.com; 3 Design and Development Department, Chongqing San Ai Hai Ling Industrial CO., Ltd., Chongqing 408100, China; wangwenbo@cqsahl.com

**Keywords:** water-lubricated bearings, bearing material, friction coefficient, wear, vibration

## Abstract

Water-lubricated bearings usually operate under severe environmental conditions, most likely in the mixed regime, in which surface contact between the drive shaft and the bearing sleeve is often significant. This presents great challenges to bearing design, especially material selection. The Tenmat, Thordon, and Rubber are common water-lubricated bearing composites. In this paper, by using a block-on-ring test apparatus, the Stribeck curve, wear rate, and vibration characteristics of three kinds of polymer materials in water-lubricated bearings (Tenmat, Thordon SXL, and Ben Teng Group (BTG) Rubber) under low speed and heavy load were studied. The experimental results show that, under the same working conditions, BTG rubber has excellent tribological properties and vibration properties. The research method in this paper can provide references for the selection of materials used for friction pair, improvement of working performance and vibration reduction of water-lubricated bearings in the future.

## 1. Introduction

Water-lubricated bearings are non-metallic bearings with water lubrication, giving them good lubrication performance and environmental friendliness. They are widely used in stern tube bearings on ships, submarines and small crafts, and hydroelectric power plants and pumps [1,2]. Water-lubricated bearing materials are usually made of bronze, iron pear wood, plastics, ceramics, nylon, polymer composites, etc. Because of the depletion of natural resources, these materials have gradually been replaced by polymer composites with some excellent performances such as a low friction coefficient and good wear resistance [3,4,5,6]. Tenmat material, widely used for high load bearings, has strong load capacity, good water lubrication property, and high reliability [7]. Thordon material is a polymer composite with high strength, corrosion resistance, and wear resistance [8]. Rubber material has a low friction coefficient, low wear, and strong deformation adaptability [9]. For example, after blending and modification of nitrile butadiene rubber (NBR) with ultra-high molecular weight polyethylene (UHMWPE), graphite acquired more outstanding low-speed performance [10]. Tenmat, Thordon, and Rubber are common water-lubricated bearing composites [11]. These three new types of water-lubricated bearing materials exhibit outstanding performance in a variety of environments, including marine and industrial environments.

However, under extreme conditions such as heavy load, low speed, start-up, and shut-down, water-lubricated bearings are prone to generate friction wear and vibration noise. Thus, friction and lubrication of water-lubricated bearing materials have always attracted attention from researchers. Wu et al. [12] and Zhao et al. [13] researched the tribological properties and wear mechanism of styrene–butadiene rubber and the polyurethane composites with dry sliding and water lubrication. Gao et al. [14] researched the tribological properties of epoxy composites at different and constant sliding speeds under water lubrication. Wang et al. [15] prepared multilayer Cr/CrN/GLC coatings on the surfaces of stainless steel rings and studied the friction and wear behaviors of the coatings on different rubbers (NBR 7201, ethylene-propylene-diene monomer (EPDM) 8370, and fluoroelastomer (FKM) 26 in the water environment. Dong et al. [16] studied the wear properties of NBR pins on 1Cr18Ni9Ti stainless steel discs in the sand water lubrication environment. Chen et al. [17] studied the effect of water medium on the tribological properties of a carbon fiber-reinforced phenolic composite, finding that the wear resistance of the composite in seawater was much better than that in pure water. Eltayeb et al. [18] studied the tribological properties of deproteinized natural rubber (DPNR) and synthetic cis-1,4-polyisoprene rubber (IR), and the effect the addition of carbon black (CB) to these composites on the friction and wear characteristics. Unal et al. [19] compared and evaluated the tribological performance of polyetheretherketone (PEEK), ultra-high molecular weight polyethylene (UHMWPE), polyetherimide (PEI), and polytetrafluoroethylene (PTFE) under dry and lubricated conditions. From the above literature, it is clear that the tribological performance of water-lubricated bearing materials varies under different working and environmental conditions. In addition, the tribological properties also depend on mechanical properties, contact geometry, load, speed, working environments, and material combination [20].

Compared with the metallic multilayer plain bearing, the water-lubricated composite bearing has the characteristics of shock resistance and vibration reduction. However, under low speed and heavy load conditions, the water film cannot be effectively formed between the shaft and the bearing. The lubrication state of water-lubricated bearings is usually mixed lubrication or even dry friction [21,22,23], which causes declining bearing capacity, serious wear, elevated temperature, and abnormal vibration. Bhushan [24] carried out an experimental research on the vibration noise mechanism of water-lubricated rubber bearings, and the results showed that the stick-slip on rubber surface is the source of vibration noise. Peng et al. [25,26] considered that the vibration noise is related to the contact area, friction coefficient, and speed of the shaft and bearing. Because of complexity and randomness, friction vibration can be easily affected by various factors. Therefore, the vibration of water-lubricated bearings must be considered, and reasonable working conditions should be investigated to suppress friction vibration. Therefore, it is necessary to screen out suitable materials and working conditions for water-lubricated bearings to reduce friction, wear, and vibration.

In this paper, the Stribeck curve, wear rate, and vibration characteristics of three kinds of materials (Tenmat, Thordon SXL, and BTG Rubber) are tested by the comparative test. The tribological properties and vibration characteristics of three kinds of block-on-ring specimens under low speed and heavy load were investigated. The experimental method provides technical support for the development of new materials for water-lubricated bearings.

## 2. Experiment 

### 2.1. Materials and Samples

**Figure 1:**
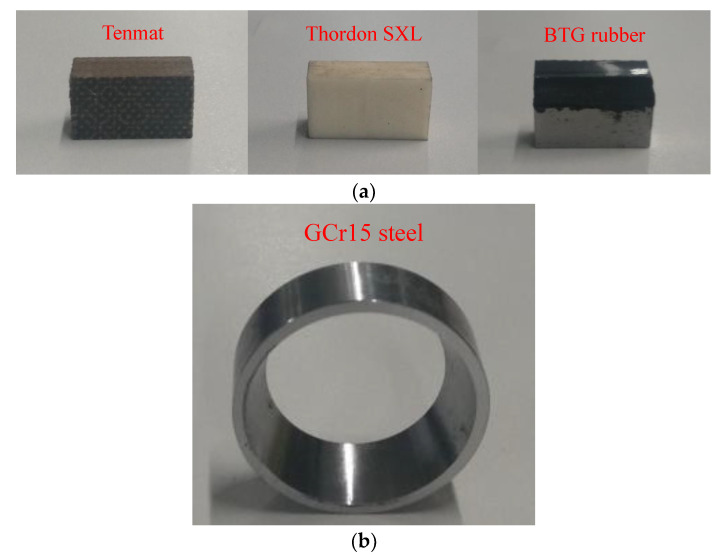
Photographs for ring and block samples. (**a**) Block specimens. (**b**) Ring specimen.

The test specimens of this study are ring and blocks. As shown in Figure 1, the ring is made of a GCr15 bearing steel with the size of 35 mm in external diameter and 10 mm in width. The blocks are the water-lubricated bearing materials, i.e., Tenmat, Thordon SXL, and BTG Rubber, which are produced by Tenmat Ltd (Manchester, UK)., Thordon Bearings Inc (Burlington, ON, Canada), and Chongqing Benteng Technology Development CO., Ltd (Chongqing, China)., respectively. The hardnesses of the three materials are 99, 97, and 64 HA, respectively. The dimensions of friction surface on test block are 15.8 mm × 6.4 mm.

### 2.2. Experimental Setup

**Figure 2:**
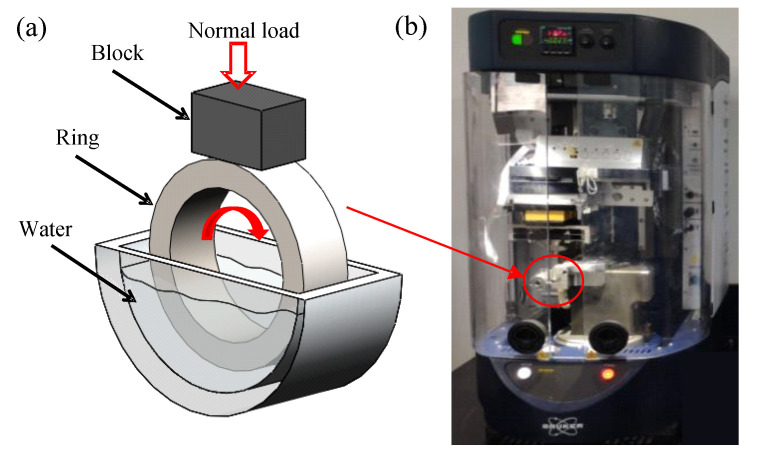
The schematic diagram of the block-on-ring module: (**a**) block-on-ring friction pair and (**b**) photograph for a block-on-ring test apparatus.

The tribological tests are carried out on the UMT-TriboLab ring-on-block tribometer (Bruker Nano Inc., San Jose, CA, USA), which is illustrated in Figure 2. This tribometer is equipped with rotational drive, and it provides the rotational motion in the vertical direction. The friction pair consists of a ring in a vertical plane and a fixed block placed horizontally, forming a line contact in between. During the tests, the upper block specimen applies a normal load that remains a constant, while the ring below slides against the surface of the block with a rotational motion. The ring specimen is submerged in lubricants. The average sliding friction coefficient is calculated by recording the normal force (Fz) and friction force (Fx) through the sensor simultaneously. The friction coefficient is equal to the ratio of the normal force to friction force.

To explore their vibration characteristics, with the three-directional sensor fixed on the loading bar, the vibration dates of three materials are obtained by the data acquisition instrument and accelerometer. The vibration acceleration amplitude is recorded by the data acquisition instrument.

### 2.3. Experimental Procedures

The effects of sliding speeds and applied loads on the tribological properties of block (Tenmat, Thordon SXL and BTG Rubber) and ring (GCr15) were studied by two sets of tribological tests under the condition of tap water lubrication, which follows the method and procedures of the ASTM G77-2017 International Standard, and the tests are carried out at room temperature with a relative humidity of 50% ± 10%. Before the test, all samples were washed in an ultrasonic bath with acetone for 15 min and then dried with a hairdryer. We replaced the lubricant after each test to avoid the effect of temperature on the tests. In order to obtain the boundary or mixed lubrication, high normal loads and low speeds are employed in the tests. In one group, the speeds of the ring vary to be 50, 100, 200, 400, and 800 rpm, and the corresponding velocities are 0.09, 0.18, 0.36, 0.72, and 1.44 m/s, respectively. The measured friction coefficient is automatically recorded by the computer. Another group is used to research the effect of different applied loads on tribological properties. The applied loads are set to 60, 90, 120, 150, and 180 N, and the corresponding maximum Hertzian pressures are 1.19, 1.46, 1.68, 1.88, and 2.06 MPa, respectively. The sliding velocity is 0.18 m/s. After tribological tests, the wear mechanism under water-existing conditions with different loads and velocities is investigated. The wear surface morphologies are observed by Contour GT-K optical profiler (Bruker Nano Inc., Tucson, AZ, USA), and the wear volume ΔV of test specimens are examined using the optical profiler. The wear rate K (mm^3^/Nm) of the test block is calculated by the following equation [27],

(1) $$K=\frac{\Delta V}{\mathrm{PL}}$$

where P represents the applied load (N) and L represents the sliding distance (m).

## 3. Result and Discussion

### 3.1. Analysis of Friction Coefficient

**Figure 3:**
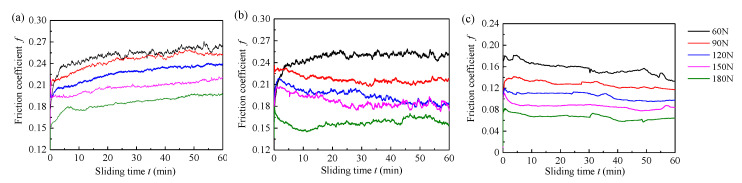
Variation of friction coefficients for three materials as a function of sliding time under tap water lubrication (different applied loads and constant velocity 0.18 m/s): (**a**) Tenmat, (**b**) Thordon SXL, and (**c**) BTG rubber.

**Figure 4:**
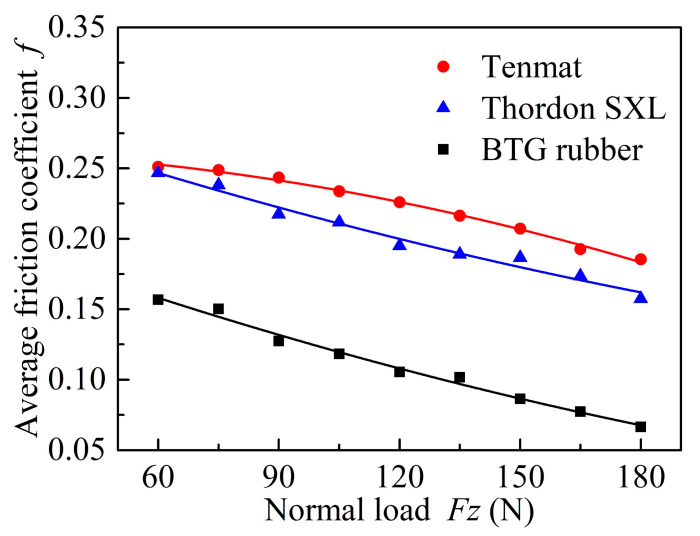
Variation of average friction coefficients for three materials under tap water lubrication (different applied loads and a constant velocity of 0.18 m/s).

Figure 3 shows the friction coefficient of three materials (Tenmat, Thordon SXL, and Rubber) when sliding at a constant speed of 0.18 m/s under tap water lubrication. It can be seen that the friction coefficients are unstable at the beginning, and then gradually flatten out after 5 min. This is probably because the equipment just started and the friction pairs have experienced the stick-slip process. For the Tenmat material, as shown in Figure 3a, the friction coefficient gradually increases with time, and the fluctuation is relatively large. This may be caused by the increasing contact area, as the wear volume of the block sample increases during the whole wear process, resulting in an increased friction coefficient and instability. Figure 3b shows that the friction coefficient of Thordon SXL seems to be relatively stable throughout the test. The friction coefficient is slightly similar at 60 and 90 N, but it decreases regularly when the applied load increases to 120, 150, and 180 N. Figure 3c shows that the friction coefficient of BTG rubber seems to be stable with no significant fluctuation, and gradually decreases in the range of 60 to 180 N [28]. This occurs because the hardness of the BTG rubber material is small, and it is more prone to elastic deformation, which is beneficial to generate the local water film and form part of the elastohydrodynamic lubrication. At the same time, the friction coefficient hardly changes over time due to the small friction loss. To study the effect of load on the friction coefficients of the three materials, the effect curve of the average friction coefficient and load is drawn, as shown in Figure 4. It is clear that the normal loads have obvious effects on the friction coefficient of the three materials. It could be seen that at the constant sliding speed, the average friction coefficient of the three materials gradually decreases with increasing normal loads. This occurs because the increase in the friction force of the three materials is less than the increase in the load, so the friction coefficient is reduced.

**Figure 5:**
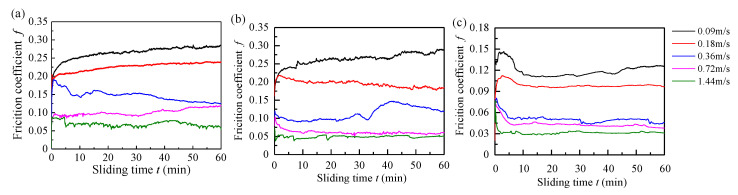
Variation of friction coefficients for three materials as a function of sliding time under water-lubricated condition (different sliding velocities and the constant applied load of 120 N): (**a**) Tenmat, (**b**) Thordon SXL, and (**c**) BTG rubber.

**Figure 6:**
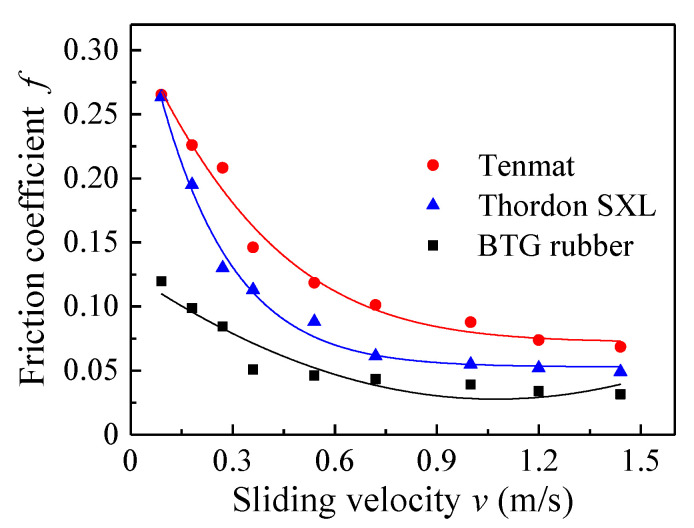
Friction coefficients for three materials under water-lubricated condition (different sliding velocities and the applied load of 120 N).

Figure 5 shows the variation of the friction coefficients for three materials as a function of sliding time, which is at a constant load of 120 N and different sliding velocities under tap water lubrication. The results show that the friction coefficient is basically stable after 10 min. The friction coefficients of the three materials vary greatly. The friction coefficient of the BTG rubber material is the smallest, Thordon SXL is the second smallest, and Tenmat is the largest. The friction coefficients of the three materials are at a higher level at the speeds of 0.09 and 0.18 m/s. Figure 6 shows the variation of the average friction coefficient of three materials as a function of sliding velocity under the normal load of 120 N with tap water lubrication. It can be seen that the change of the average friction coefficient versus sliding speed meets the Stribeck curve with normal load. This shows that the friction coefficients of the three materials decrease rapidly at first and then bottom out. This can be explained by the fact that the water film appears with an increased speed, and therefore the friction coefficient decreases. At a sliding speed of less than 0.3 m/s, it is difficult to form an effective water film. It is worth noting that in the speed range of 0.36 to 1.44 m/s, the water film thickness gradually increases and pressure increases, resulting in a significant decrease of the friction coefficient [12]. The friction coefficient of rubber material is the lowest at low speed, which indicates that it is easier to form the lubricating water film at low speed. The rubber material is more suitable for low-speed operation. With the increase of speed and load, the friction coefficients of the Thordon and Tenmat materials gradually become close to that of rubber material. It is not difficult to infer that with the continuous increase of speed and load, the friction coefficients of Thordon and Tenmat materials will be lower than that of the rubber material, indicating that Thordon and Tenmat materials are more suitable for high speed and heavy load working conditions [29].

### 3.2. Analysis of Wear Volume at Different Velocities

**Figure 7:**
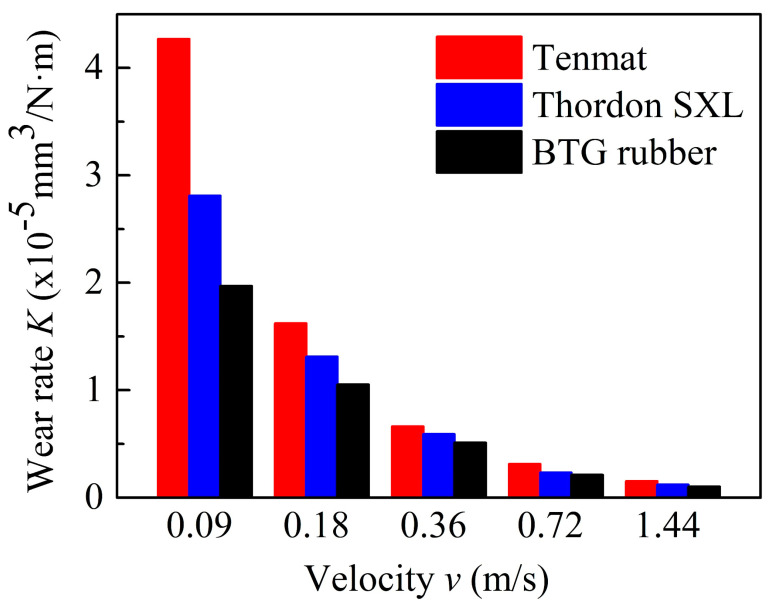
Wear rate curves of three materials at different velocity (Fz = 120 N).

**Figure 8:**
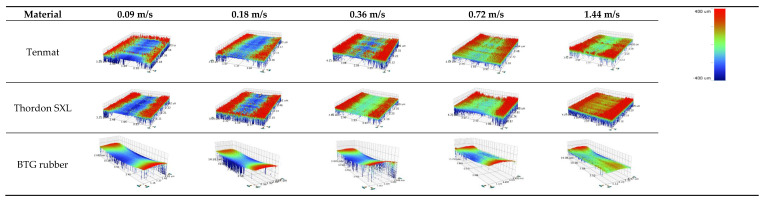
3D optical profile images of the wear tracks for three materials under different velocities.

To measure the wear rate of three kinds of water-lubricated bearing materials, the wear volume loss of the block specimen was measured by 3D Optical Profiler. Figure 7 shows the wear rates of three water-lubricated bearing materials at different operating speeds with a constant normal load of 120 N under water lubrication. As can be seen in this figure, all the wear rates decrease with increasing speeds. Furthermore, the wear rates of three materials follow the order of Tenmat > Thordon SXL > Rubber at the same test condition. The 3D optical images of wear traces confirm this in Figure 8. This occurs because the lubrication state of the friction pairs of the three materials has changed with the increase of speed. The lubrication state of the friction pair changes from mixed lubrication to elastohydrodynamic lubrication. The contact of the micro-convex body of the friction pair decreases gradually, the friction force decreases, and the wear amount decreases. This is essentially consistent with the results in Chen [17]. Moreover, at low speed, the wear rate of BTG rubber is the lowest because of the lowest friction coefficient. With increasing velocity, the friction coefficient and wear rate of the three materials become closer.

### 3.3. Analysis of Wear Mechanism

**Figure 9:**
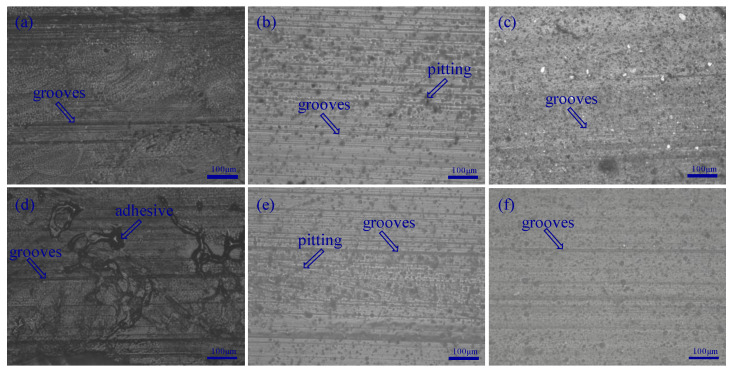
Microscopy of block worn surfaces: (**a**) Tenmat, (**b**) Thordon SXL, and (**c**) BTG rubber at 0.09 m/s (applied load 120 N); (**d**) Tenmat, (**e**) Thordon SXL, and (**f**) BTG rubber at 1.44 m/s (applied load 120 N).

The wear surfaces of the blocks under the tap water lubrication are examined by 3D Optical Profiler to study the wear mechanism. Figure 9 shows the microscopy images of the wear tracks for three different blocks at constant sliding velocities of 0.09 m/s and 1.44 m/s under water lubrication with a normal load of 120 N. As shown in Figure 9, there are many scratches on the worn surface along the sliding direction. This is due to the plowing effect of the hard asperities of the GCr15 ring against the soft polymer surface, signifying a considerable amount of abrasive wear [30]. Therefore, for the materials and experimental conditions in this study, the abrasive wear runs from the beginning to the end. The sliding generates heat at the contact area, which tends to soften the polymer. This is a sign of the presence of an adhesive wear mechanism [2]. In the experimental conditions of this study, the wear mechanism is a combination of adhesive and abrasive wear for Tenmat [31]. As shown in Figure 9b and e, there are many grooves and pittings on the worn surface along the sliding direction. Signifying a considerable amount of abrasive wear and fatigue wear. Therefore, the wear mechanism is a combination of fatigue and abrasive wear for Thordon SXL [32]. Few smooth scratches are observed on the wear surfaces of BTG rubber, so its wear mechanism is abrasive wear.

### 3.4. Analysis of Vibration

**Figure 10:**
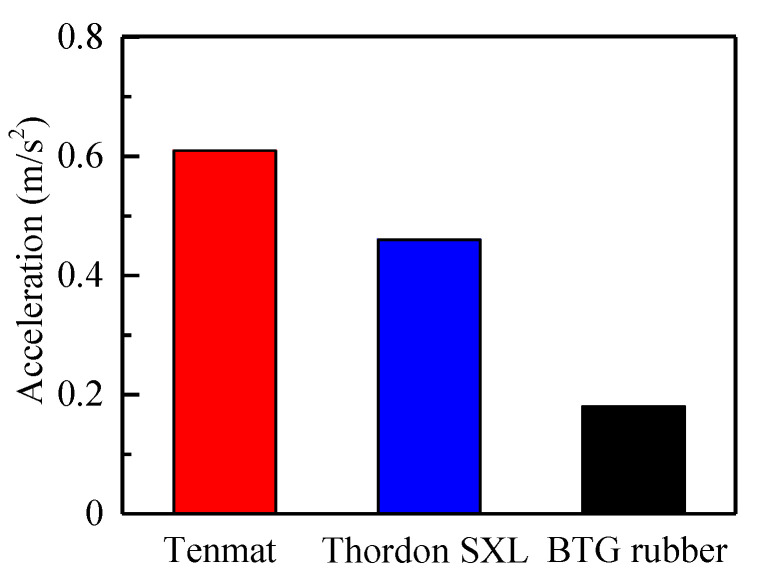
Vibration acceleration amplitude of three materials at normal load 60 N and sliding velocity 0.09 m/s.

**Figure 11:**
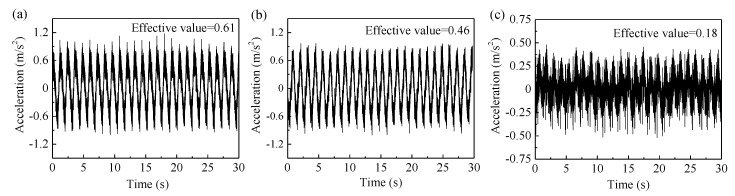
The time domain curve of the (**a**) Tenmat, (**b**) Thordon SXL, and (**c**) BTG rubber.

At low speed and heavy load conditions, the water-lubricated bearings often generate abnormal vibration [33]. This may be due to the stick-slip caused by the failure to form a lubricating water film between the shaft and bearing. Stick-slip is the main factor that generates friction vibration for the water-lubricated bearings [34]. This paper analyzes the representative vibration in the Z direction. Figure 10 shows the vibration acceleration of Tenmat, Thordon SXL, and BTG rubber in the Z direction under the normal load of 60 N and sliding velocity of 0.09 m/s. It can be seen that the acceleration amplitudes of Tenmat, Thordon SXL, and BTG rubber are 0.61 m/s^2^, 0.46 m/s^2^, and 0.18 m/s^2^, respectively. The vibration amplitude of the BTG rubber is much smaller than that of the other two materials. This is because the secondary force existing in the molecular structure of rubber produces viscoelasticity, leading to a good shock absorption performance. Besides, friction is the root cause of vibration [35], so the continual change of the frictional force causes the change of vibration amplitude [36,37]. The increase in vibration in turn increases friction and wear rate. The three influence each other [38]. Therefore, it can also be seen from Figure 11 that the amplitude of vibration acceleration has the same trend as the friction coefficient and wear rate. They follow the order of BTG rubber < Thordon SXL < Tenmat. Therefore, friction reduction, wear reduction, and noise reduction should be considered together, instead of changing a single performance.

## 4. Conclusions

In order to guide the selection of water-lubricated bearing materials and clarify the performance of corresponding materials, the relationships between friction, wear rate, and vibration were explored by comparing the properties of three common water-lubricated bearing composites under different working conditions. The friction, wear, and vibration of three common water-lubricated bearing materials (Tenmat, Thordon SXL, and BTG rubber) under water lubrication were compared. By analyzing the results of the friction coefficient, wear rate, wear mechanism, and vibration, this study draws the following conclusions.

(1) The tribological test results demonstrate that the friction coefficients, wear rate, and vibration of the three materials follow the order of Tenmat > Thordon SXL > BTG rubber with the same test conditions. The friction coefficients and wear rates of the three materials decrease with increasing speed and applied load, among which the speed has a greater impact on the tribological performance of the three materials than that of the applied load. The results show that the elastohydrodynamic lubrication is easier to be formed by increasing rotating speed compared to increase load.

(2) For the test conditions of this investigation, the wear mechanisms of Tenmat, Thordon SXL, and BTG rubber are a combination of adhesive and abrasive wear, a combination of fatigue and abrasive wear, and abrasive wear, respectively.

(3) The friction coefficient, wear rate, and vibration of the rubber material are the lowest at low speed, which indicates that it is easier to form the lubricating water film at this time. The rubber material is more suitable for low-speed operation. With increasing speed and load, the friction coefficient of the Thordon and Tenmat materials gradually becomes closer to that of the rubber material. It is not difficult to infer that with the continuous increase of speed and load, the friction coefficient of Thordon and Tenmat materials will be lower than that of rubber material, indicating that the Thordon and Tenmat materials are more suitable for high speed and heavy load working conditions.

(4) Compared with Tenmat and Thordon SXL, BTG rubber shows a much smaller vibration amplitude and better shock absorption performance. The friction is the root cause of vibration, so the continual change of the frictional force causes the change of vibration amplitude. The increase in vibration in turn increases friction and wear rate. The three influence each other. Therefore, friction reduction, wear reduction, and noise reduction should be considered together instead of changing a single performance.
